# For Sustainable Career Development: Framework and Assessment of the Employability of Business English Graduates

**DOI:** 10.3389/fpsyg.2022.847247

**Published:** 2022-04-14

**Authors:** Minjun Tong, Tianyue Gao

**Affiliations:** ^1^School of Foreign Languages and Business, Minjiang Teachers College, Fuzhou, China; ^2^School of Economics, Hebei University, Baoding, China

**Keywords:** employability, sustainable career development, business English graduate, mixed methods research, gender difference

## Abstract

Employability is an important indicator of the competency of the employees. Employability model is a useful analytical framework for studying the ever-changing relationship between higher education and the job market. At present, the demand for business English graduates is increasing, however, there is a skill gap between their educational readiness and the recruitment requirements. In order to solve this problem, this study adopted mixed methods research and carried out the research design according to the exploratory sequence design to construct an employability model for business English graduates. A 46-item scale was developed to measure the employability of business English graduates’ employability. After assessment, it was found that the employability of business English graduates was multi-dimensional, with three dimensions—professional knowledge, generic competencies, and career management and 10 sub-dimensions—English language skills, foreign trade competencies, computer and internet application skills, social skills, learning and development, personal traits, thinking ability, work ethics, career identity and planning, and service awareness. This study verified that the employability of business English graduates reached the standard of talent training and met the requirements of employers. There were employability differences in gender, places of origin and educational institutions. From this study, it could be inferred that cultivation of business English majors should be multi-dimensional.

## Introduction

With the internationalization of the world economy, economy integration has become the mainstream of present world development which brings increasingly frequent commerce and business events. The sustained growth of foreign trade and the increasing number of foreign trade companies have created large number of employment opportunities, which has led to an increasing demand for foreign trade practitioners. Talents in the field of foreign trade who are good at foreign business communication and understand international trade rules are becoming the focus of higher education.

However, many employers mention a common problem that many graduates are not competent for jobs after graduation because they cannot combine the theoretical knowledge with the practical operation of the job (Helle et al., 2006). Many graduates have difficulty in employment because of their weak career competency which is important in their career development. Career development has traditionally focused on acquiring work abilities and earning experience in a certain position (Akkermans et al., 2013). Career development encompasses a wide range of problems, including the growth of talents, the preservation of present skills, and the preparation for the future following promotion (Kaya and Ceylan, 2014). A sustainable career is dynamic and adaptable, with continual learning, periodic renewal, the security of employability, and a harmonic match with personal abilities, interests, and values (Newman, 2011). Dynamic careers have grown more frequent in recent decades, with people progressing through horizontal moves across various organizations (De Vos et al., 2021). In order to get and keep a job in this changing labor market, individuals need professional competencies to help them manage their careers (Heijde and Van Der Heijden, 2006). Employability is essential in a sustainable career, which is the capacity to keep one’s current job or obtain new work as needed (Donald et al., 2018).

Grotkowska et al. (2015) pointed out that internationally, higher education providers are shifting from its role as an “ivory tower” concept to a “market-oriented education enterprise.” Employability plays an important role in this concept. Recent changes in education and labor market policies have increased the demand on higher education institutions to cultivate employable graduates, although what are the elements of employability and what attributes of graduates are needed to cultivate are still controversial (Bridgstock, 2009). Employability skills are skills that are critical to a company’s or industry’s growth and development (Husain et al., 2012). Employability allows employees or graduates to succeed in a variety of circumstances. Employability is a key condition for an organization to maintain a competitive edge and for individuals to attain career satisfaction. Stimulating employees’ vocational skills and employability seems to be good for both organizations and the employees’ outcomes (Fugate et al., 2004; Van Dam, 2004). High-employability workers (Van Dam, 2004) are essential for organizations to meet fluctuating demands for quantitative and functional flexibility (Marginson, 1989; Valverde et al., 2000).

Researchers have noted that there is a “skill gap” between employment requirements and the educational readiness of graduates (Morley, 2001; Andrews and Wooten, 2005). Specifically, employers do not believe that higher education has successfully developed the employability skills of graduates (Peddle, 2000). One of the reasons is that college teaching is out of line with social needs, and there is a lack of an effective employability assessment system in its measurement.

Several studies have been done on employability. Some of the research is about the concept and definition of employability, among which the most well-known are Hillage and Pollard’s (1998), Yorke and Knight’s (2004), McQuaid and Lindsay’s (2005), and Pool and Sewell’s (2007). Some scholars proposed their understandings of employability (Fugate et al., 2004; Heijde and Van Der Heijden, 2006; Rodrigues et al., 2019). Some scholars put forward the framework of employability and analyzed the factors affecting employability. Hillage and Pollard’s framework of employability was the most thorough one around 2000, which greatly inspired the studies of subsequent scholars on employability (Hillage and Pollard, 1998). Other scholars’ employability models, such as McQuaid and Lindsay’s framework (McQuaid and Lindsay, 2005), USEM model (Yorke and Knight, 2004), CareerEDGE model (Pool and Sewell, 2007) and some other models, all provided a good model foundation for employability research. Some scholars have done research on model construction of employability. Abelha et al. (2020) discussed the graduate employability and competence development in higher education. Albina and Sumagaysay (2020) discussed the employability of information technology education graduates. However, there was little discussion on the employability of business English graduates, which is of great importance in the talent cultivation to enhance the employment status of business English graduates. Therefore, this study would discover and explain the following issues. First, what components are necessary in the employability of business English graduates? Second, how can the employability of business English graduates be measured? Third, what are the results of assessment? In this study, the employability framework of business English graduates was constructed, and an employability measurement tool was developed. After that, the employability scale was used to assess the employability of the recent business English graduates. A comprehensive set of employability measurement tool allows individual employees, especially the newly graduates, to keep track of their abilities and career requirements. Therefore, the framework and assessment tool of the employability of business English graduates provides a guarantee for employers and business English graduates joining the workforce to meet the changing needs of modern enterprises, and get sustainable career development.

## Literature Review and Theoretical Background

### Employability

The concept of employability has been subjected to the social and historical swings of each period, and has adopted a wide variety of meanings. Employability is the ability to find the first job, keep employment and be able to get a new job when needed (Hillage and Pollard, 1998). Employability is also used to imply the attributes of graduates, which individuals own and can prove that they are capable to hold a job (Harvey, 2000). Employability refers to the competency required and embodied in the work position. It is a collection of understandings, achievements and personal qualities, increasing the likelihood of graduates finding jobs and progressing in their chosen career, which benefits themselves, the workforce, society, and the economy (Knight and Yorke, 2003). Employability is a complex of skills, knowledge, understanding and personal attributes, which helps an individual to easily choose and keep an occupation in which he is satisfied and can get some achievements (Pool and Sewell, 2007). Rae (2007) believes that the employability of college graduates is a combination of a series of personal development achievements such as knowledge, skills and personality traits acquired by students through study and practice. These results enable college graduates to adapt to job requirements, achieve employment faster and succeed in their chosen career. This concept affirms that employability of college students is the result of higher education and the potential for the development and success of college students in their future work. Nabi (2003) points out that employability is that graduates have the right skills and attributes and can use them to gain and maintain appropriate employment opportunities. It can be defined in various ways, but it means obtaining characteristics that ensure employment (Harvey, 2000), which can not only ensure employment, but also ensure competence and sustainable development in the job.

There seems to be a consensus that employability is not a one-dimensional construct, but is composed of parameters of a very varied nature (e.g., McQuaid and Lindsay, 2005; Heijde and Van Der Heijden, 2006; Fugate and Kinicki, 2008). Employability is of great importance to college graduates for future employment. Many scholars and researchers have built their own employability framework based on their understandings of employability.

Hillage and Pollard (1998) summarized all previous and existing ideas about employability for the first time, which is seen as pioneering. Hillage and Pollard’s (1998) employability framework consists of four interacting components of employability: “employability assets,” “deployment,” “presentation,” and “context factors.” Hillage and Pollard emphasized that employability should focus on the individual factors of job seekers. Based on Hillage and Pollard’s employability framework, McQuaid and Lindsay (2005, p. 206) provided a broader view of employability. McQuaid and Lindsay (2005) grouped and extended these employability components into three major factors: “individual factors,” “personal circumstances,” and “external factors.” The component of “individual factors” includes more elements than the other two components. The “employability skills and attributes” listed in McQuaid and Lindsay’s framework covers the competencies that an individual should acquire for the employment: “essential attributes,” “personal competencies,” “basic, key and high-level transferable skills.” Of the three components of the employability framework, the “individual factors” seems to be the most influential. The USEM account of employability (Yorke and Knight, 2004) is probably the most well-known and respected model in this field. The acronym USEM stands for four interconnected elements of employability: (1) understanding; (2) skills; (3) efficacy beliefs; and (4) metacognition. Pool and Sewell (2007) discussed the various elements of employability and developed the CareerEDGE model, which is an option that incorporated all of the important elements and employability skills in the USEM models. They believed that Career Development Learning, Experience (Work and Life), Degree of Knowledge, Generic Skills and Emotional Intelligence were the five components at the lower level of the model. The model provided clear information about what needed to be considered and what was included.

In fact, employability is a dynamic concept. It continues to evolve and change with the requirements of economic and social development. In different social stages, the connotations and requirements of employability are different. In this study, the components of employability would be focused on individual level, which seemed to be the most influential (McQuaid and Lindsay, 2005).

### Requirements of Competences for Business English Graduates

Different stakeholders proposed different requirements of competences for business English graduates. The employers believe that, in order to improve students’ overall quality, personal cultivation, morality, English proficiency, business knowledge and operational skills should have an organic combination (Kim, 2015). Overseas-client-development ability and business English language proficiency are essential career competences. Besides, the abilities of product cognition, business negotiation, computer operation, cost accounting, billing and settlement, contract control and signing, logistics operation, business tracking, etc., are also necessary. The core competitiveness of business English graduates is reflected in two aspects, one is good business English proficiency and the other is foreign trade knowledge (Kim, 2015). The abilities that business English graduates should possess are mainly attributed to three areas: learning ability, communication skills and teamwork spirit (Glaser, 1994). Some companies mention professional quality, which is also an aspect of career competences, such as business professional skills, communication skills, teamwork ability, professional attitude, crisis management ability, learning ability, interpersonal communication and expansion ability. Besides these abilities, comprehensive quality and work ethics are also valued by the enterprises.

Teachers believe graduates should master both English language ability and business practice ability, have international vision, cross-cultural communication ability and speculative ability, and be able to be engaged in international business work (Walker, 2009). Ciortescu and Cecal (2015) believe that business English graduates should possess skills, cross-cultural awareness and effective communication, especially the core abilities that are helpful for future development. Cross-cultural awareness, social skills and negotiation skills are also necessary for business English graduates, as they will communicate and negotiate in a multi-cultural environment in the future. In the global business field, cross-cultural awareness is a must for any business person. Professional knowledge ability refers to language ability and practical ability, while social skills refer to the abilities to take on certain social responsibilities and to deal with interpersonal relationship properly, so as to get development in the future career. Social skills include a wide range of abilities, including environmental adaptability, organizational management, interpersonal communication, teamwork, decision-making, innovation, and some other abilities. In the syllabus designed for business English students, language, business, cross-cultural communication and humanistic literacy are emphasized (Derado, 2015). Besides language, business knowledge and business skills, humanistic knowledge and intercultural communication skills are also needed for business English graduates (Kim, 2020).

Based on the above, hypothesis 1 can be proposed: The employability of business English graduates is multi-dimensional, composed of different dimensions.

### Employability and Higher Education

One of the functions of education is to provide a skilled workforce (Rich, 2015). Employability of graduates has been clearly recognized as one of the main objectives of higher education (Sumanasiri et al., 2015). Employers and policy makers expect higher education to improve students’ employable skills (Asonitou, 2015). Cole and Tibby (2013) believe that there is a strong consensus that employability is not just about obtaining employment, and that HEPs should not focus solely on supporting students to obtain their first job, but on supporting them to build active and meaningful careers and to participate meaningfully in society. Higher education can attract and retain high-quality students (Hewitt-Dundas and Roper, 2018) and train high-quality graduates to meet the growing demand for the workforce (Boden and Nedeva, 2010). Artess et al. (2017) argue that employability provided by higher education should be multi-dimensional, experiential, and embedded in the curriculum, institutional processes and provisions. Although higher education plays an important role in employability, there are also some impractical curricula that hinder students’ employability and cause a graduate skills gap (Tran, 2018). So the employability of young graduates is a very important criterion to evaluate the results of the teaching activity in HEIs (Şerban, 2013).

It can be inferred from previous research that cultivating graduate employability is the task of higher education. HEIs hope that the quality of talents they cultivate is to achieve the goal of training and meet the needs of employers. Thus, hypothesis 2 can be proposed: The employability of business English graduates reaches the objective of talent training and meets the needs of employers.

### Assessment of Employability

Both changes in the workplace and new demands are reflected in the way employability is assessed, and the assessment must clearly reflect the nature of the required skills (Saterfiel and McLarty, 1995). As with the framework of employability, there are various scales that try to evaluate employability. Daniels et al. (1998) developed a Perceived Employability Scale (PES) to measure self-efficacy in the individual’s working trajectory, which considers four factors: interpersonal efficacy; information gathering and barrier removal efficacy; persistence; and goal-setting efficacy. This scale is a five-point Likert scale with 15 items, testing adults with different cultural, ethnic and racial backgrounds. Rothwell and Arnold (2007) measured employability from a self-perceived perspective, developing a self-perceived employability scale from two dimensions-the internal and external dimension and the four quadrants. The questionnaire includes three factors: (a) personal factors, concerning attitudes and personal work-related competences, like personal qualities such as responsibility or social skills; (b) socio-personal factors, referring to socio-familial variability (the search of the first job) and social labor market and job position according to gender, age, educational level and activity sector. In the research by Yusof et al. (2012), employability skills had nine dimensions: critical thinking and problem-solving skills; pursuit of lifelong learning and information management skills; communication skills; teamwork; technical skills; entrepreneurship; leadership; ethics and social responsibility. Jiang et al. (2013) developed a consistent measurement framework based on the definition of the concept of college students’ employment, and conducted relevant empirical tests. They believed that college students’ employability was a multi-dimensional construct consisting of four independent factors: general professional ability, professional ability, professional supporting skills and personality traits.

From the previous research, it could be inferred that employability can be measured and affected by gender and different background, such as family background and educational background. Thus, hypotheses can be proposed as follows:

Hypothesis 3: There is gender difference in the employability of business English graduates.

Hypothesis 4: There is difference in the employability of business English graduates from different place of origin.

Hypothesis 5: There is difference in the employability of business English graduates from different institutions.

## Methodology

**FIGURE 1:**
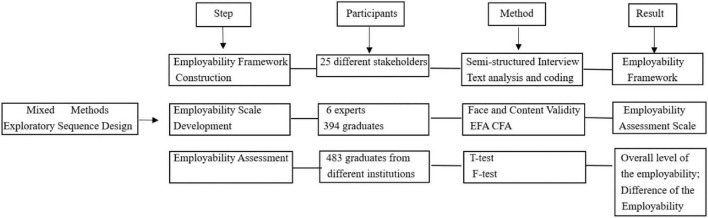
Procedure of this study.

This study adopted Mixed Methods research and carried out the research design according to the exploratory sequence design, which combined qualitative research and quantitative research. In qualitative research, the first step was to draft the interview outline based on the previous research (see Appendices I–IV). Qualitative research was conducted to explore the elements required for the employability of business English graduates, so participants for qualitative interviews were recruited by purposive sampling, who were all related to business English talents. Twenty five different stakeholders (six senior foreign trade practitioners, six junior foreign trade practitioners, six employers, and seven business English teachers) received individual in-depth interviews in different ways. Face to face interviews were used with senior foreign trade practitioners and junior foreign trade practitioners, telephone interviews were used with two employers, a face-to-face interview was held with one senior teacher and video interviews were used to collect data of other senior teachers. After the in-depth interview, encoded and analyzed the interview data, and constructed an initial employability framework. Finally, drew up the questionnaire according to the coding results of the interview data. Six experts were invited to verify the face validity and content validity. In quantitative research, a pilot test was performed to test the reliability and validity of the questionnaire, and SPSS 22 and AMOS 21 were used to conduct exploratory factor analysis (EFA) and confirmatory factor analysis (CFA). After the validation, a model of 3 dimensions and 10 sub-dimensions was constructed, and a 46-item scale of employability for business English graduates was developed. In the questionnaire, participants were asked to rate how aware they were of their skills and knowledge, such as “I understand the culture and customs of different countries and pay attention to cultural differences when communicating with clients.” “I can do market research and be sensitive to market trends.” etc. The Likert scale was coded 1–7, where 1 was assigned to “very disagreeable” up to 7 meaning “very agreeable.” These numbers were used to symbolize which level of awareness to which the graduate related. The formal assessment was carried out by purposive sampling, and SPSS 22 was used to analyze the quantitative data. In the quantitative research, recent graduates of business English and those who had just graduated within 3 years were recruited from nine different types of higher vocational education institutions in Fujian, China. Three hundred and ninety-four effective participants were recruited in the pilot test and 483 effective participants were recruited in the assessment. The procedure of this study is shown in Figure 1.

## Data Analysis

### Data Analysis of the Interview

**FIGURE 2:**
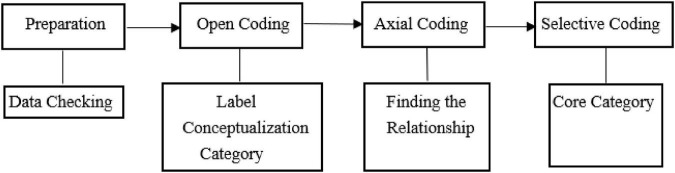
Process of interview data analysis.

The coding method of grounded theory was used in this study. The data was analyzed according to the procedures of open coding, axial coding and selective coding. The data was analyzed line-by-line and combined to develop dimensions, categories and themes which led to a larger and more comprehensive understanding of what was examined. Data analysis in this study began as soon as the participants and researcher started interacting, and it continued throughout the interviews until the data was saturated. The point at which no new relevant information emerge is referred to as saturation (Yin, 2014). NVIVO 12 software was used to assist data analysis, which can help organizing, sorting, coding, and categorizing large amounts of data and can identify and compare patterns and themes from various perspectives. Figure 2 shows the process of interview data analysis.

Firstly, the data was carefully examined line by line to eliminate distortion, doubt and content that might be against the common sense. Secondly, in open coding, the ideas, implications and meanings of the text were revealed. The data was decomposed into individual parts, and then the similarities and differences were examined and compared in detail, the events or cases were grouped with similar concepts or associated meanings into more abstract concepts to discover and establish categories. The most powerful “meaning unit” in the text was selected as the coding reference point, and each coding reference point was numbered, such as “S-01.” Thirdly, on the basis of open coding, the relationship between various concepts or categories was found, so as to show the relationship between each other. Fourthly, selective coding was conducted which was the process of integration and further refinement of the theory. Through the systematic analysis and comparison of all the discovered concepts and categories, a dominant “core category” was selected, which could pull all the other categories into a whole, playing the role of “summarization.” NVIVO12 software was used for data management and comparative analysis of the data. After interview analysis, a framework of employability for business English graduates was obtained (as shown in Table 1).

**TABLE 1:** Employability framework of business English graduates.

High-order construct	First-order construct	Items
Professional knowledge	English language skills	English reading ability
		Oral English communication ability
		English correspondence writing ability
		English translation ability
		Cross-cultural knowledge and awareness
		English terminology
	Foreign trade competencies	Familiarity with the products
		Professional knowledge
		Basic knowledge of foreign trade
		Business negotiation ability
		Customer development and tracking
		Reasonable quotation
	Computer and internet application skills	Computer elementary operation
		Internet application
		Operation on foreign trade platforms
Generic competencies	Social skills	Teamwork
		Communication and coordination
		Communication and expression
		Interpersonal communication
	Learning and development	Quick learning
		Active learning
		Lifelong learning
		Sustainable development
	Personal traits	Stress resistance
		Aesthetic ability
		Patience and attentiveness
		Confidence
		Self-motivated
	Thinking ability	Analysis, thinking and making judgment
		Creative thinking
		Analyze and solve problems
		Crisis management
Career management	Career attitude	Keep promise
		Professionalism
		Loyalty
		Sense of responsibility
		Career and work planning
		Industry interest
	Service awareness	Initiative service
		Empathy
		Treat customers sincerely

### Data Analysis of Pilot Test

Three hundred and ninety-four valid questionnaires were collected in this pilot test. One hundred and fifty samples were randomly selected for EFA, and the remaining 244 samples were used for CFA and validity test.

#### Exploratory Factor Analysis

The employability framework obtained by the Qualitative Research was an evaluation structure consisting of three first-level dimensions and nine second-level dimensions. The three first-level dimensions were professional knowledge, generic competencies and career management ability, while the nine second-level dimensions were English language skills, foreign trade competencies, computer and internet application skills, social skills, learning and development, personal traits, thinking ability, career attitude and service awareness. In this factor analysis, the KMO value was 0.904 > 0.7, and Bartlett’s sphericity test result was *p* < 0.001, which means the scale was suitable for principal component analysis.

Three first-level dimensions could be extracted, of which the first factor explanation rate is 37.631% < 40%, and there are no serious common method biases in the questionnaire. The load value of each item and corresponding dimension is higher than 0.5. In addition, the combined reliability (CR) value of each dimension of the questionnaire is higher than 0.8, and the average variance extraction (AVE) is higher than 0.5, so the questionnaire has good convergent validity and discriminant validity. As mentioned above, the scale was a two-level-dimension scale. The value of KMO was 0.941 > 0.7 in the dimension of professional knowledge ability, which could extract three secondary dimensions with the factor loading of each item higher than 0.5, CR value higher than 0.8, and AVE higher than 0.6; the value of KMO was 0.886 > 0.7 in the dimension of generic competencies, which could extract four secondary dimensions with the load value of each item higher than 0.5, CR value higher than 0.8 and AVE higher than 0.4; the value of KMO was 0.821 > 0.7 in career management ability, which could extract three secondary dimensions with the load value of each item higher than 0.5, CR value higher than 0.8 and AVE higher than 0.6.

The results above showed that the questionnaire could be divided into 3 first-level dimensions and 10 second-level dimensions, and the validity of the questionnaire met the analysis requirements. The reliability values of the three first-level dimensions were all higher than 0.7, and the reliability results of the second-level dimensions were also higher than 0.7. In summary, the results of this questionnaire survey were of high stability and acceptable reliability, and the questionnaire items were of good quality.

The total correlation coefficient of each item of the questionnaire is higher than 0.3, and the reliability value after deleting each indicator is not higher than 0.961, so all the items should be retained. After analyzing the variance, it was found that the *F*-value is 19.955, and the corresponding *P*-value is 0.000 < 0.05, indicating that the items of the questionnaire had basic discriminating power.

**FIGURE 3:**
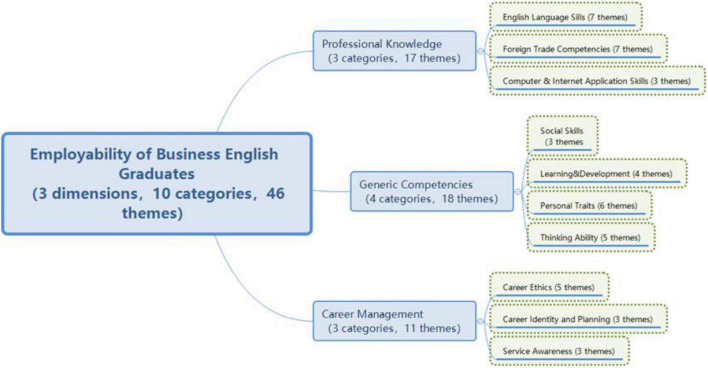
Revised framework after exploratory factor analysis.

After EFA, the framework of three second-level dimensions and nine first-level dimensions obtained from the qualitative research were modified into a framework of three second-level dimensions and 10 first-level dimensions (as shown in Figure 3).

In this study, through qualitative data analysis and exploratory factor analysis, it was found that the employability of business English graduates consisted of different dimensions, thus verified Hypothesis 1: The employability of business English graduates is multi-dimensional, composed of different dimensions.

#### Confirmatory Factor Analysis

The Cronbach’s alpha coefficient of the overall reliability of the questionnaire was 0.974, which was basically consistent with the reliability of the exploratory factor analysis, of high stability. The reliability calculation results of each dimension also met the analysis requirements (as shown in Table 2).

**TABLE 2:** Reliability of each dimension.

Second-order dimension	First-order dimension	No. of items	Cronbach’s alpha	
Professional Knowledge	English Language Skills	7	0.943	0.963
	Foreign Trade Competencies	7	0.958	
	Computer and Internet Application Skills	3	0.841	
Generic Competencies	Thinking Abilities	3	0.935	0.968
	Personal Traits	4	0.934	
	Learning and Development	6	0.941	
	Social Skills	5	0.956	
Career Management Ability	Work Ethics	5	0.941	0.960
	Career Identity and Planning	3	0.912	
	Service Awareness	3	0.920	

**FIGURE 4:**
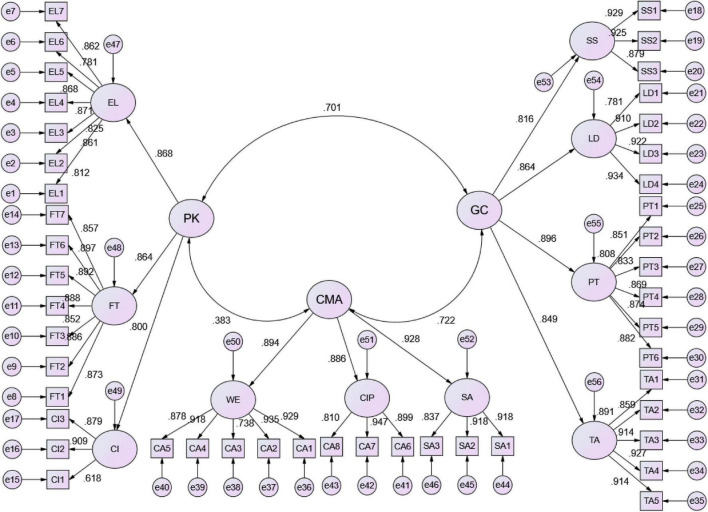
Result of confirmatory factor analysis.

Confirmatory factor analysis results are shown in Table 3 and Figure 4. Each item and the second-order factor loading, regression coefficients of the second-order factors and the first-order factors were all higher than 0.5, the CR value was higher than 0.6, and the calculation results were in line with the requirements. Besides, the measurement model demonstrated acceptable model fit of the data. A number of measures generated by AMOS were also used to evaluate the goodness-of-fit of the measurement model including the ratio of chi-square/degrees of freedom, Tucker-Lewis index (TLI), incremental fit index (IFI), comparative fit index (CFI), standardized root mean square residual (SRMR) and Root Mean Square Error of Approximation (RMSEA). The ratio of chi-square/degrees of freedom is 2.67, which is below the desired value of 5.0 as recommended by Schumacker and Lomax (2004). The TLI and IFI values are 0.912 and 0.920, respectively, indicating a good fit (Bentler, 1989; Hair et al., 2010). Further, CFI (0.919), SRMR (0.05), and RMSEA (0.066) are within the acceptable levels.

**TABLE 3:** Factor loadings and composite reliability.

Factor	Indicator	Standard factor loading	SE	*z*-Value	*p*-Value	CR	AVE
EL	EL1	0.812	0.051	19.011	0.000	0.944	0.706
	EL2	0.860	0.048	20.789	0.000		
	EL3	0.826	0.048	19.511	0.000		
	EL4	0.871	0.049	21.237	0.000		
	EL5	0.868	0.048	21.133	0.000		
	EL6	0.780	0.052	17.921	0.000		
	EL7	0.862	0.050	20.905	0.000		
FT	FT1	0.873	0.052	21.397	0.000	0.959	0.771
	FT2	0.885	0.048	21.915	0.000		
	FT3	0.850	0.050	20.512	0.000		
	FT4	0.890	0.047	22.097	0.000		
	FT5	0.893	0.048	22.228	0.000		
	FT6	0.896	0.049	22.379	0.000		
	FT7	0.857	0.050	20.788	0.000		
CI	CI1	0.636	0.055	13.319	0.000	0.853	0.664
	CI2	0.902	0.052	21.783	0.000		
	CI3	0.879	0.052	20.918	0.000		
SS	SS1	0.930	0.042	23.638	0.000	0.936	0.830
	SS2	0.927	0.042	23.502	0.000		
	SS3	0.876	0.045	21.388	0.000		
LD	LD1	0.780	0.044	17.936	0.000	0.937	0.789
	LD2	0.912	0.040	22.977	0.000		
	LD3	0.922	0.041	23.420	0.000		
	LD4	0.932	0.040	23.871	0.000		
PT	PT1	0.849	0.046	20.392	0.000	0.941	0.728
	PT2	0.808	0.045	18.863	0.000		
	PT3	0.831	0.046	19.695	0.000		
	PT4	0.871	0.045	21.241	0.000		
	PT5	0.877	0.043	21.470	0.000		
	PT6	0.881	0.041	21.658	0.000		
TA	TA1	0.892	0.042	22.190	0.000	0.956	0.812
	TA2	0.860	0.044	20.878	0.000		
	TA3	0.914	0.042	23.149	0.000		
	TA4	0.928	0.042	23.746	0.000		
	TA5	0.911	0.041	23.012	0.000		
WE	WE1	0.927	0.039	23.740	0.000	0.946	0.779
	WE2	0.935	0.039	24.077	0.000		
	WE3	0.736	0.051	16.575	0.000		
	WE4	0.919	0.041	23.351	0.000		
	WE5	0.880	0.041	21.671	0.000		
CIP	CIP1	0.903	0.044	22.447	0.000	0.917	0.786
	CIP2	0.943	0.039	24.174	0.000		
	CIP3	0.811	0.048	18.906	0.000		
SA	SA1	0.918	0.041	23.094	0.000	0.921	0.796
	SA2	0.917	0.041	23.034	0.000		
	SA3	0.839	0.044	19.917	0.000		
PK	EL	0.868	0.053	15.784	0.000	0.882	0.713
	FT	0.864	0.056	17.030	0.000		
	CI	0.800	0.052	11.071	0.000		
GC	SS	0.816	0.047	17.210	0.000	0.917	0.734
	LD	0.864	0.044	15.451	0.000		
	PT	0.896	0.048	17.669	0.000		
	TA	0.849	0.045	17.514	0.000		
CMA	WE	0.894	0.043	19.590	0.000	0.930	0.815
	CIP	0.886	0.047	18.382	0.000		
	SA	0.928	0.043	20.145	0.000		

*EL, English Language Skills; FT, Foreign Trade Competencies; CI, Computer and Internet Application Skills; SS, Social Skills; LD, Learning and Development; PT, Personal Traits; TA, Thinking Abilities; WE, Work Ethics; CIP, Career Identity and Planning; SA, Service Awareness; PK, Professional Knowledge; GC, Generic Competencies; CMA, Career Management Ability.*

### Data Analysis of the Assessment

The Assessment Scale for Business English Graduates’ Employability was used to assess the employability of business English graduates, so as to explore the level of their employability, and to see whether there were significant differences in different demographic variables, such as different family background, different institutions and different genders.

#### The Overall Level of the Employability of Business English Graduates (Test of Hypothesis 2)

Hypothesis 2 of this study was that the employability of business English graduates meets the standard of talent training and the requirements of employers. Since the scale in this study was a 7-point Likert scale, when the score was 4, it indicated that the conformity was “ordinary.” Therefore, if the average score of the total scale was greater than 4, it implied that the participants’ employability reaches the standard of talent training and meets the requirements of the employers.

In order to test Hypothesis 2, this study conducted a one-sample test on all the samples. It was found that the significance of English Language Skills (EL) was 0.456 > 0.05, and the other nine factors were significant, *p* = 0.000 < 0.05. The 95% confidence interval of the difference showed that the lower limit of English Language Skills (EL) was negative, and the upper and lower limits of the other nine factors were all positive. Therefore, it could be inferred that except for the factor English Language Skills (EL), the mean value of the other nine factors affecting the employability of business English graduates was all >4, among which the mean value higher than 5 were factors Social Skills (SS), Learning and Development (LD), Personal Traits (PT), Work Ethics (WE), Career Identity and Planning (CIP) and Service Awareness (SA). The average value of English Language Skills (EL) is 4.0446, reaching the general level. Therefore, it could be inferred that Hypothesis 2 was true, and the employability of business English graduates reaches the standard of talent training and meets the requirements of employers. Among the employability skills, English Language Skills (EL) basically meets the requirements and needs to be further strengthened.

In order to test the specific abilities of business English graduates in various aspects of English Language Skills (EL) factor, this study conducted statistical analysis on each variable of EL factor. The mean value of EL2 (I have good English listening ability and can communicate with the clients in fluent spoken English.), EL4 (I can communicate with clients by correspondence in good English.), EL5 (I can do English-Chinese translation accurately.) was less than 4, and the mean value of EL6 (I know the cultures and customs of different clients’ countries, and I will pay attention to the cultural differences in my communication with clients.) included four items. It could be inferred that the graduates need to improve their skills in English listening and oral communication, foreign trade correspondence, and translation.

#### Gender Difference in Employability of Business English Graduates (Test of Hypothesis 3)

Hypothesis 3 of this study was that there is gender difference in the employability of business English graduates. According to the results of the Levene’s test of homogeneity of variance, except for the *F*-value of the Learning and Development (LD) factor, which was 0.022, there were no significant differences between the male and female in the variance of other nine factors. The significance of English Language Skills (EL) was 0.001 (*p* < 0.05), and the significance of Foreign Trade Competencies (FT) was 0.004 (*p* < 0.05), while the significance of the other eight factors that affect employability were all greater than 0.05 (*p* > 0.05). Therefore, among the 10 factors that affect the employability of business English graduates, there was no significant gender difference in the other eight influencing factors except for English language Skills (EL) factor and Foreign Trade Competencies (FT) factor. It could be inferred that Hypothesis 3 was true. There is gender difference in the employability of business English graduates, which is mainly in “English language skills” and “foreign trade competencies.” In addition, after the *post-hoc* test, it was found that the mean values of male in English Language Skills (EL) and Foreign Trade Competencies (FT) was higher than those of female.

#### Difference in the Employability of Graduates From Different Places of Origin (Test of Hypothesis 4)

Hypothesis 4 of this study was that there is difference in the employability of graduates from different places of origin. After one-way ANOVA analysis, it was found that among the 10 factors that affect employability, the significance of Work Ethics (WE) was 0.028 (*p* < 0.05), and that of the other nine factors was all greater than 0.05 (*p* > 0.05). That was to say, among the factors affecting the employability of business English graduates, there was difference of student origin in Work Ethics (WE) factor, while there was no significant difference in the other nine factors. Since there was significant difference in Work Ethics (WE), after the *post-hoc* test, it was found that the graduates from cities and counties had significant difference in the mean value of Work Ethics (WE), while there was no significant difference between graduates from cities and towns and villages in Work Ethics (WE), and the mean value of the work ethics of graduates from the city was higher than those from the county. It could be inferred that Hypothesis 4 was true. There is difference in the employability of different student origin, mainly in “Work Ethics.”

#### Difference in the Employability of Graduates From Different Institutions (Test of Hypothesis 5)

Hypothesis 5 of this study was that there is difference in the employability of graduates from different institutions. After one-way ANOVA analysis, among several factors that affect employability, the *F*-value of Foreign Trade Competencies (FT) was 3.423, *p* = 0.033 (<0.05), the *F*-value of Learning and Development (LD) was 3.585, *p* = 0.028 (<0.05), and the *F*-value of Work Ethics (WE) was 4.359, *p* = 0.013 (<0.05), while the significance of other influencing factors was all greater than 0.05 (*p* > 0.05). In other words, among the ten influencing factors, there were differences in different institutions in the factors of Foreign Trade Competencies, Learning and Development, and Work Ethics, while the other seven factors showed no difference in different institutions. Due to the significant differences in Foreign Trade Competencies, Learning and Development and Work Ethics, after *post hoc*, it was found that there were significant differences in Foreign Trade Competencies (FT) and Work Ethics (WE) between graduates of national demonstrative vocational colleges and graduates of ordinary vocational colleges. The mean value of Foreign Trade Competencies (FT) and Work Ethics (WE) of graduates from the national demonstrative vocational colleges was higher than that of ordinary vocational colleges; while there were significant differences in “Learning and Development” and “Work Ethics” between graduates of national demonstrative vocational colleges and graduates of provincial demonstrative vocational colleges. The mean value of “Learning and Development” and “Work Ethics” of graduates from national demonstrative vocational colleges was higher than that of graduates from provincial demonstrative vocational colleges. It can be inferred that Hypothesis 5 was true. There is difference in the employability of graduates in different institutions, mainly in “Foreign Trade Competencies,” “Learning and Development,” and “Work Ethics.”

## Discussion

### Employability Is Multi-Dimensional and Measurable

In this study, the framework of employability for business English graduates was obtained through mixed-method research. Based on the characteristics of the post, this study interviewed different stakeholders to determine the competences required by the job, including knowledge, skills and abilities, so as to judge the individual’s competence for the post, namely, the individual’s employability. This framework contained three first-level dimensions and 10 sub-dimensions, indicating that the employability of business English graduates is a set of abilities composed of different dimensions, which is consistent with the previous view that “employability is not a single-dimensional structure, but consists of parameters with very different properties” (McQuaid and Lindsay, 2005; Fugate and Kinicki, 2008). It’s a multi-faceted concept with a series of achievements, including skills, understanding and personal traits. In this framework, 10 sub-dimensions are included, covering the core employability that can be widely adapted and qualified for different job requirements and can be transferred across different setting, as well as professional ability that meets specific industry or job requirements. To have good employability, in addition to mastering the professional skills required by the job, one should also have the generic competences, so as to be competent for the current position and get development in the career. Employability is defined as “skills required not only to gain employment, but also to progress within an enterprise to achieve one’s potential and contribute successfully to enterprise strategic directions” (Department of Education, Science and Training [DEST], 2002, p. 3).

Employability can be measured, but firstly the concept must be operationalized. Operationalization is the process of going from a theoretical concept to a measurable index (Harvey and MacDonald, 1993). In the survey, it was found that the employability of business English graduates meets the standards of talent training, but there is still much room for improvement; there are some differences in gender, place of origin and higher education institutions.

### The Cultivation of Business English Majors Should Be Multi-Dimensional

People must be employable both quickly and sustainably in a constantly evolving environment, which necessitates not only the acquisition and mastery of the skills and knowledge unique to their own profession or career, but also the possession of generic skills, dispositions, and qualities that are transferable to occupational situations and fields. The cultivation of employability is not just “transfer of skills,” but it is connected to graduate identity (Hager and Hodkinson, 2009). Therefore, in the process of talent cultivation, HEIs should prioritize the diversified cultivation of students’ abilities. Employability is measured in different dimensions, which also indicates that the cultivated talents should have multi-dimensional abilities to address the needs and growth for the society. In the study, it was found that some skills and qualities are still in need of enhancement, and there are some differences in the employability of graduates from different institutions, which may have been caused by the curriculum setting and teaching input of the colleges.

Validation processes and module learning outputs can make employability explicit in classes. As in most fields of study, effective experience in delivering student employability within the program necessitates the use of a combination of learning and teaching methods, including lectures, and individual and group projects. Successful learning, training, and evaluation projects can help students make strong, well-founded employability (Knight and Yorke, 2003, p. 3).

In the development of student employability, it is also worth noting that graduates should take time to learn and invest in some essential graduate attributes, such as leadership, teamwork and coordination. Specific skills should be identified within disciplinary fields and employers place a strong emphasis on “soft skills,” which are difficult to describe, distill, or communicate (Mourshed et al., 2012, p. 67). Thus, it should be a trend for universities to offer “additional” employability modules as part of degree programs, focusing on the development of “soft skills” or “core competencies” in areas such as negotiation and influence, communication, teamwork or presentation. Career management is also important in employability skills. Career management should start as early as students receive higher education, which should be obligatory and credit-bearing in academic programs (Bridgstock, 2009). HEIs should also notice that for the students receiving higher education, it seems more important to help them learn and raise confidence, self-esteem and ambitions rather than just focusing narrowly on abilities and skills (Pegg et al., 2012).

Generally speaking, course curricula should be helpful for employability development. With the diversification of college types, student sources, characteristics, and socio-economic backgrounds, HEIs should also adjust the cultivation of students’ employability. HEIs should not cultivate technical personnel with narrow vision and inflexibility, but must attach importance to the positive desire to learn and be able to reflect and criticize in order to cultivate talents who are adaptable, innovative and capable of leading progress. This is in line with the philosophy of university education.

## Limitations and Future Research

Several limitations are discussed as follows. First, this study was about the employability of business English graduates. The samples were recruited from different types of colleges in Fujian Province. These samples were not sufficient to represent the nationwide situation. It is recommended to expand the scope of sampling and recruit more participants from different provinces in future research. Second, comparative studies could be conducted from the process of talent cultivation to find out the reasons for the differences in the employability of graduates from different institutions while the study compared the results of the employability assessment of graduates. Third, the factors of employability should be diversified, including both internal and external factors, while this study focused primarily on internal factors that graduate employability without considering the impact of external factors. In future research, the influence of external factors on employability could be studied.

## Data Availability Statement

The raw data supporting the conclusions of this article will be made available by the authors, without undue reservation.

## Author Contributions

MT contributed to the conceptualization, investigation, and methodology. MT and TG contributed to the formal analysis, validation, writing—original draft, and writing, reviewing, and editing. Both authors contributed to the article and approved the submitted version.

## Conflict of Interest

The authors declare that the research was conducted in the absence of any commercial or financial relationships that could be construed as a potential conflict of interest.

## Publisher’s Note

All claims expressed in this article are solely those of the authors and do not necessarily represent those of their affiliated organizations, or those of the publisher, the editors and the reviewers. Any product that may be evaluated in this article, or claim that may be made by its manufacturer, is not guaranteed or endorsed by the publisher.

## Appendix

**Appendix I:**

Interview outline for junior foreign trade practitioners.	
1	What was your biggest confusion when you first started doing foreign trade? (Follow up and ask for specific examples.)
2	Is there any work experience that impressed you? How did you deal with it?
3	What do you think is the most important ability to do a good job in foreign trade? Can you give some practical examples?
4	How do you feel about your performance? (Why it’s good, or why it’s not good enough)
5	Is the knowledge learned at college useful at work? How does it connect with the work?
6	What knowledge and abilities do you think colleges should teach so that you can adapt more quickly when you enter the foreign trade industry?
7	Why do you think the top performers in your company do well? Can you give specific examples?
8	What are the main reasons for underperforming people in your company? Can you give specific examples?
9	What are the advantages and disadvantages of being male (female) in foreign trade industry? Can you give some specific examples?

**Appendix II:**

Interview outline for senior foreign trade practitioners.	
1	Why did you choose to work in the foreign trade industry?
2	Is there any experience at work that impressed you in particular? How did you deal with it? Could you tell me more about it?
3	What kind of abilities do you think you possess so that you can be in this industry for so long?
4	What skills are needed to do a good job in foreign trade? Can you give some examples?
5	In what ways do you improve your ability and make yourself better in the foreign trade industry?
6	There are many business English graduates entering the foreign trade industry now, are there around you? What skills do they need to improve? Can you give specific examples? How do you think it can be improved?
7	Do you think there are gender differences in the foreign trade industry? Who is more suitable for this industry, male or female? Or, who will do better?

**Appendix III:**

Interview outline for employers.	
1	What abilities do companies value when recruiting employees? Are these competencies necessary for the job? Why?
2	Are there any employees who have particularly impressed you? Can you tell me more about it?
3	What qualifications do you think a person should possess in order to take root and develop in foreign trade industry?
4	How long does it take for fresh graduates to adapt to the job when they first enter the workforce? (Why does it take so long?)
5	Is there any gap between the teaching and training in the college and the requirements of your company? What are these gaps mainly reflected in?
6	Does your company have a gender bias in employee recruitment? Why?
7	Do you have any comments or suggestions for colleges in talent cultivation?

**Appendix IV:**

Interview outline for senior business English teachers.	
1	How many students does your business English major enroll each year? What about the employment situation of the graduates?
2	What employability do you think a graduate should have in order to find a suitable job in foreign trade?
3	Does the college take these aspects into account in its curriculum? What courses are offered to develop their employability?
4	Have you found any abilities in which students are relatively weak?
5	Do you think the capabilities of business English graduates match the requirements of enterprises? What improvements need to be made?
6	Does the college have rules and regulations for teachers to go to enterprises for further training? Can you tell us about it?
